# Draft Whole-Genome Sequence of the Alkane-Synthesizing Polar Cyanobacterium Pseudanabaena biceps Strain O-153

**DOI:** 10.1128/MRA.00904-20

**Published:** 2020-11-19

**Authors:** Bérangère Péquin, Julien Tremblay, Christine Maynard, Jessica Wasserscheid, Charles W. Greer

**Affiliations:** a McGill University, Department of Natural Resource Sciences, Ste-Anne-de-Bellevue, Quebec, Canada; b Energy, Mining and Environment Research Centre, National Research Council Canada, Montreal, Quebec, Canada; Portland State University

## Abstract

Alkane biosynthesis by polar cyanobacteria has not yet been reported. We present here the draft whole-genome sequence of an alkane-synthesizing polar cyanobacterium, Pseudanabaena biceps strain O-153. The genes coding for the two key enzymes involved in the alkane biosynthetic pathway were found contiguously in the genome.

## ANNOUNCEMENT

Cyanobacteria from different environments are known to synthesize alkanes (1, 2). However, cyanobacteria from extreme environments are less known due to challenges in isolating and growing these microorganisms. Here, we report the genome sequence of a polar cyanobacterial strain, labeled O-153, isolated from Bylot Island (Canadian Arctic) in an epilithic pond (73°20′00″N, 78°47′20″W) in 1993 by W. Vincent (Université Laval, Québec, Canada), who kindly provided us with this strain. The sample was grown in liquid BG11 medium (3) and then isolated on the same medium on a 2% agar plate (4). The strain was grown in the laboratory in liquid BG11 medium with incubation at 14°C and a 12:12-h light/dark cycle with irradiation at 5 to 28 μE m^−2^ s^−1^. Microscopically, strain O-153 appeared as a thin blue-green filament, forming a short chain (3 to 5 cells), adhering slightly to glass. It is nonmotile, 1.9 by 2.5 μm with a volume of ∼7.5 ± 4.2 μm^3^, living in a mixed culture with heterotrophic bacteria. Morphologically, it was identified as being in the *Oscillatoriales* order.

DNA was extracted using the Ultraclean microbial kit (Qiagen). A short-read library was prepared using the Qiaseq FX DNA library kit (Qiagen) and sequenced on a MiSeq system (2 × 250 bp) using v2 chemistry (Illumina). A 20-kb SMRTbell library was prepared and sequenced on one single-molecule real-time (SMRT) sequencing cell on a PacBio Sequel system using v3.0 chemistry (Pacific Biosciences) at the Génome Québec Innovation Center (McGill University, Montréal, Canada).

The *de novo* assembly was carried out using the Hierarchical Genome Assembly Process (HGAP.4) (5) using SMRT Link v6.0.0. The coverage cutoff was set to 30× and the estimated genome size to 5 Mbp. Raw subreads (1,350,044 subreads) having a read quality value of <0.7 (pbcoretools v1.5.0) were left out, and remaining subreads (164,880) were used as input for the Falcon assembler (Falcon kit v1.2.2). Assembly polishing was performed with Arrow (v2.2.2) to give a total of 49 contigs representing 5,523,450 bp. The polished assembly was scaffolded using SSPACE-Longread (6) with default parameters. To further correct for sequencing artifacts, MiSeq sequencing data, generated with the same starting DNA, were aligned against the scaffolds (BWA v0.7.17). Consensus scaffolds were generated with BCFtools v1.9 (7), giving 34 linear scaffolds totaling 5,569,051 bp with an *N*_50_ value of 3,970,957 bp consisting of one 3,970,956-bp chromosome and eight plasmids ranging from 8,252 to 239,714 bp and a G+C content of 44.8%. Plasmids were identified with PlasFlow v1.1 (8) and were not found to be circularized. Final corrected scaffolds were annotated using the NCBI Prokaryotic Genome Annotation Pipeline (9). The chromosome assigned to Pseudanabaena biceps (using fastANI v1.31 [10]) contains 4,906 coding sequences (CDS), 3 rRNA genes (16S, 23S, and 5S rRNAs), 4 noncoding RNAs, and 57 tRNA genes. This uncircularized chromosome assembly has a range similar to those of other known *Pseudanabaena* genomes (11). The genome was estimated to be highly complete at 99.06% (CheckM v1.0.4 [12]). The genes coding for the alkane biosynthetic pathway’s two enzymes were detected contiguously in the annotated genome.

### Data availability.

This whole-shotgun project has been deposited in GenBank under the accession number JAATWA000000000 (assembly GCA_013361095.1). Raw reads are available under the BioProject accession number PRJNA615646 and BioSample number SAMN14463795 (SRR12827764 for PacBio reads and SRR12827763 for Illumina reads).
